# Multifunctional PCL/Lignin-PCL Composite Films for Delivery of Atrazine and Metribuzin for Sustainable Agriculture Applications

**DOI:** 10.1021/acsagscitech.5c00081

**Published:** 2025-06-18

**Authors:** Alvaro G. Garcia, Omar E. Mendez, Fannyuy V. Kewir, Gabriel D. Patterson, Artur Klamczynksi, Onu Onu Olughu, Carlos E. Astete, James D. McManus, Cristina M. Sabliov

**Affiliations:** † Biological & Agricultural Engineering, 5779 Louisiana State University and LSU Ag Center, Baton Rouge, Louisiana 70803, United States; ‡ Bioproducts Research Unit, 1096 WRRC, ARS-USDA, Albany, California 94710, United States

**Keywords:** lignin, poly(ε-caprolactone), grafting, film, metribuzin, atrazine, release, herbicide

## Abstract

Sustainable agriculture calls for the development of eco-friendly materials that possess desired properties and functionalities. In this study, the effect of incorporating lignin-grafted poly­(ε-caprolactone) (LN-PCL) into a PCL matrix was evaluated. LN-PCL was synthesized by ring-opening polymerization (ROP), yielding polymers with varying PCL degrees of polymerization (DP 26–101) and amphiphilic properties. Incorporating LN-PCL into PCL films enhanced hydrogen bonding, crystallinity, and doubled Young’s modulus. SEM analysis showed smoother surfaces with higher DPs, while lower DPs reduced the contact angle from 78° to 68°. LN-PCL films absorbed >98% UVAB and >94% UVC light regardless of DP and degraded within 30 days. Release studies indicated controlled release rates of ATZ (<16%) and MTZ (46%) over 10 weeks. Overall, the UV protection, surface, mechanical, and controlled release properties of the LN-PCL/PCL films support the potential of these films as a carrier for chemicals with applicability in agriculture.

## Graphical abstract

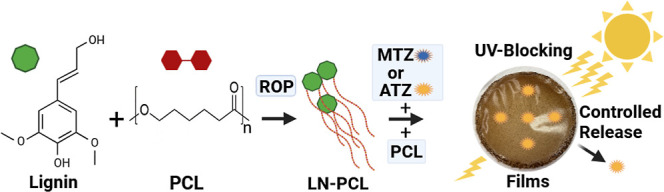

## Introduction

Lignin (LN), the second most abundant natural polymer, provides mechanical support and protection against pathogens within plants.^1^ LN is generated in the amounts of 50 to 70 million tons annually worldwide^2^ as biowaste in various industries such as pulp and paper, agriculture, and biorefineries.^3^ Due to its chemical structure and modifiability, LN finds uses in a variety of applications. For example, the complexity and chemical framework of lignin structure can provide rigidity to composites when used as reinforcement,^4,5^ and the abundance of hydroxyl groups in LN (e.g., carboxyl, phenolic and aliphatic) facilitates modification and chemical attachment of other polymers. Despite LN’s potential to yield functional materials, it is treated as low-cost waste and is mostly used in heat generation or as animal feedstock. However, through modification, LN can be valorized to create high-value polymers with diverse applications in biomedicine, agriculture, and food packaging.^6^

Blending or grafting LN with other polymers offers several advantages. Functionalization of LN modulates the solubility of LN-based polymers in organic solvents > depending on the chemistry, and with the correct modifications, it can form an amphiphilic polymer with surfactant properties.^7,8^ Grafting of LN onto biodegradable polymers allows the creation of matrices^9–11^ that could be used as delivery systems for agrochemicals. Moreover, LN-modified polymers demonstrate enhanced thermal properties and photostability, attributable to phenolic and ketone functional groups that provide UV light absorbance and coloration.^12,13^

It is therefore not surprising that LN-based nanostructures were developed as biodegradable alternatives with controlled-release agriculture applications, aiming to reduce active ingredient loss and environmental contamination while utilizing renewable materials.^14^ Several studies showed that LN-based nanoparticles offer improved release, stability, and efficiency of nanodelivered agrochemicals.^10,15–21^ In our group, polycaprolactone (PCL) and poly­(lactic-*co*-glycolic acid) (PLGA) were grafted to LN and further formed into nanoparticles.^9,10^ The LN-based nanoparticles with negative zeta potential and a size ranging between 100 and 300 nm exhibited controlled release of insecticides in aqueous solutions^10,19^ and facilitated the translocation of the agrochemicals to plant tissue without adverse effects on soybean plants at concentrations below 0.2 mg/mL.^19,20^

Similarly, incorporation of LN into films has been explored by our group and others as reinforcement of polymeric matrices focusing on enhancing the thermal and physical properties of the structures. Previous studies reported on the characteristics of composite films made from polymers modified by the addition of LN showed that the addition of LN improved the films’ mechanical properties while modulating their degradation, which is beneficial for agricultural applications that call for longer release times.^22,23^ Further, by using an interphase formation technique, functional LN-based amphiphilic polymers, formed by grafting LN to PLGA, in this case, alkali (ALN) and lignosulfonate lignin (SLN), were assembled into films with two sides of differing characteristics: one side hydrophobic and the other side polar.^24^ In another study, LN-based films were shown to be effective in coating urea-based fertilizers, extending the fertilizer’s release times in comparison to traditional polymeric films.^25^ Additionally, nanoformulations of herbicides have demonstrated increased efficacy, achieving superior weed control compared to conventional formulations at 10-fold the recommended dose.^26^ Similarly, nanoformulations using biodegradable nanocarriers, such as lignin, zein, and chitosan, exhibit reduced toxicity.^27^ These findings suggest that controlled-release systems with biodegradable carriers can enhance weed control efficacy while mitigating the negative impacts on crops and soil health. Specifically, for long-residual herbicides like atrazine, our LN-PCL/PCL films aim to reduce carryover phytotoxicity risks by controlling release rates, potentially lowering soil persistence compared with conventional formulations.

To date, no study has explored the incorporation of LN-PCL into PCL films for herbicide delivery systems. This work introduces a novel approach by developing eco-friendly LN-PCL/PCL composite films that integrate controlled herbicide release with UV protection, offering a sustainable alternative to nonrenewable materials in short-lived agricultural products. We evaluated two widely used, long-residual herbicides, metribuzin (MTZ, hydrophilic) and atrazine (ATZ, hydrophobic), chosen for their extensive agricultural use, persistent environmental impact, and contrasting solubilities, which enabled us to examine diverse release behaviors from the films. These herbicides were selected to showcase the potential of biodegradable, controlled-release systems to address the environmental persistence of such compounds, a pressing challenge in modern agriculture. Although MTZ is banned in the European Union due to environmental concerns, its inclusion remains relevant for regions where it is still applied and for designing safer delivery methods that could guide future herbicide development. Likewise, ATZ’s widespread use and persistence position it as an ideal candidate for enhancing application efficiency and reducing ecological harm via slow-release technology. This study investigates how the degree of polymerization (DP) of PCL, grafted onto LN via ring-opening polymerization (ROP), influences the mechanical, thermal, and controlled-release properties of LN-PCL/PCL composite films. We hypothesized that the PCL chain length, controlled by the ϵ-caprolactone/LN (CL/LN) mass ratio during ROP, influences these properties. We report data on the mechanical, thermal, and physicochemical properties and biodegradability of the films. By correlating these properties with the release profiles of MTZ and ATZ, we aim to advance biodegradable delivery systems, reducing the dependence on toxic, persistent herbicides, and contributing to sustainable weed management practices in agriculture.

## Materials and Methods

### Materials

Polycaprolactone (PCL, *M*_n_ = 45,000 g mol^–1^), ε ε-caprolactone (CL, >99%), and stannous 2-ethyl hexanoate (Sn­(Oct)_2_, purity 92.5–100.0%) were acquired from Sigma-Aldrich (St. Louis, MO). Alkaline lignin (LN) was acquired from TCI Inc. (Portland, OR). Methanol (purity ≥99%), dichloromethane (DCM, purity ≥99.9% extra dry), toluene (purity ≥99.5%, extra dry), and deuterated chloroform (CDCl_3,_ purity ≥99.7%) were acquired from Fisher Scientific (Pittsburgh, PA).

### Synthesis of LN-PLC Grafting

The LN-PCL polymers were synthesized using a method described in a previous study.^10^ Briefly, 2 g of alkali LN was placed in a three-neck round-bottom flask (250 mL), followed by the addition of CL at specific CL/LN mass ratios (w/w). Three different CL/LN mass ratios were utilized (2, 6, and 10), which led to three different DP values (26, 57, and 101), as determined by ^1^H NMR. The resulting polymers presented different PCL chain lengths.

The reaction was held in an oil bath at 130 °C. Following the addition of 0.2% Sn­(Oct)_2_ as the catalyst (v/v based on CL volume), the reaction was allowed to proceed for 24 h under constant stirring. Next, 200 mL of cold methanol was added to remove monomers and unreacted PCL. The resulting grafted polymer was washed five times using 200 mL of DCM and distilled water to eliminate any unreacted LN. Subsequently, the polymer was concentrated, frozen, and lyophilized utilizing a freeze-dryer (FreeZone Plus 2.5) to remove residual water and solvents. Finally, the LN-PCL polymer was stored in a desiccator at room temperature to ensure its dryness and stability for future applications.

### Film Synthesis

The production of the PCL film involved a modified approach to solvent casting.^28^ The PCL/LN-PCL mass ratio was fixed at 2:1 due to the film-forming characteristics of LN-PCL. At a low degree of polymerization (DP), LN-PCL alone cannot form a stable thin film and requires blending with a higher-DP polymer, such as PCL, to ensure film formation. This 2:1 ratio was consistently applied across all films to facilitate a systematic comparison of how varying the LN-PCL DP affects the properties of the resulting PCL films. First, 600 mg of PCL pellets were dissolved in 10 mL of DCM, and then 300 mg of filler (LN-PCL_26_, LN-PCL_57_, LN-PCL_101_, or LN) was added and allowed to dissolve for 1 h. The resulting solution was poured into a 100 mm diameter polytetrafluoroethylene (PTFE) evaporating dish and allowed to dry for 48 h at room temperature. Subsequently, all samples were stored in desiccators until further use. Agrochemical loading was accomplished following the same method, and metribuzin or atrazine (90 mg) was added to the DCM.

### Chemical Characterization of Films

The confirmation of LN’s linkage to PCL was established through Fourier transform infrared (FT-IR) analysis utilizing a Bruker Tensor 27 spectrometer (Bruker 500, Billerica, MA). The assessment was performed on dried samples under 32 scan cycles at a spectral resolution of 4 cm^–1^. The spectral range scanned was between 400 cm^–1^ and 4000 cm^–1^, focusing on the evaluation of hydroxyl and carbonyl functional groups to assess the interplay between the polymers.^29^

Nuclear magnetic resonance (NMR) spectroscopy analyses were conducted using a Bruker 400 spectrometer (Billerica, MA) at a frequency of 400 Hz, employing CDCl_3_ as the solvent. For ^1^H NMR spectra, the focus was on determining the average length of the PCL arms connected to LN. This was achieved by calculating the ratio of signal peak integrals between the areas at 4.03 ppm (corresponding to repeating –CH_2_O−) and 3.65 ppm (representing terminal –CH_2_OH).^30,31^ In this study, we used LN-PCL polymers with PCL DP of 26, 57, and 101, incorporated in the PCL matrix with a DP of 103.

### Thermal and Physical Characterization of Films

Differential scanning calorimetry (DSC) analysis was conducted by using a TA Q100 DSC instrument (TA Instruments, New Castle, DE). The process involved determining the crystallization temperature (*T*_c_) and melting temperature (*T*_m_) as part of the characterization, which has been documented elsewhere.^10,32^ Approximately 2–4 mg of the individual samples of pure LN, PCL, and LN-PCL polymers were enclosed within an aluminum pan. Subsequently, a controlled heating and cooling cycle ranging from 0 to 120 °C was executed at a rate of 10 °C/min, all within a nitrogen environment.

The crystallinity of the samples was calculated according to the following equation

1 $$X_{C}\left(\%\right)=\left(\frac{{\Delta H}_{f}}{{{\Delta H}_{f}}^{*}}\right)\times 100\%$$

where Δ*H*_f_ refers to the melting enthalpy (J g^–1^) obtained from the fusion peak of DSC, Δ*H*_f_* = 136.1 J g^–1^, that is the heat of fusion for 100% crystalline PCL.^30^

The assessment of thermal stability for the individual samples of pure LN, PCL, and LN-PCL polymers was carried out by thermogravimetric analysis (TGA) using a TA TGA550 instrument from TA Instruments. Each sample, comprising 3–5 mg, was placed in an aluminum pan. Subsequently, the temperature was raised to 600 °C, employing a heating rate of 50 °C per minute, all under a nitrogen flow.^10^

Mechanical characteristics of the films were evaluated using an INSTRON universal testing system series 5969 (Norwood, MA). A tensile test was conducted at a consistent head speed of 12.5 mm/min.

Several parameters were determined, including the maximum tensile strength (MPa), the yield strength (MPa), the elongation at break (%), and Young’s modulus (GPa). The polymer films were sliced into strips of approximately 10 × 30 mm. Measurements of width, length, and thickness were taken using a micrometer. Tensile strength (measured in MPa) and strain (measured in mm) were extracted from the resulting tensile strength curve.^24^

### Surface Characterization of Films

The water-repellent properties of the films were evaluated using a sessile drop test using methodology from another study.^24^ Briefly, the contact angle (CA) was measured with an optical tensiometer (Attention Theta, manufactured by Biolin Scientific, Beijing, China). For the measurement, a 15 μL drop of distilled water (DI) was placed on the film’s surface and allowed to remain there for 30 s before recording the contact angle; this was done for both sides of the film.

The structural characteristics of film surfaces were examined using scanning electron microscopy (SEM), following previously published methodology.^24^ The film samples were coated for 8 min with platinum (Pt) and securely affixed to double-sided carbon tape. A FEI Quanta 3D FEG dual-beam FIB/SEM microscope (FEI/Thermo Fisher, Waltham, MA) was employed to capture the SEM images. Detection was facilitated by an EDAX Apollo XL EDS detector (EDAX/AMETEK, Mahwah, NJ).

### Optical Properties

UV light transmittance of films was measured using UVC (220 to 275 nm) and UVAB (280 to 400 nm) digital light meters from General tools & Instruments (New York, NY), under illumination by a PortaRay 400W Arc Lamp UVB with a UV light (400W, 280–315 nm) from Uvitron International Inc. (West Springfield, MA). The UV transmittance was measured for all films at 27.9 ± 0.6 μW/cm^2^ and 54.5 ± 0.7 μW/cm^2^ for UVAB and UVC, respectively (*n* = 3).

### Degradation Analysis of Polymers

Organic compost containing plant material and food waste (WonderGreen Compost, American Soil & Stone, Richmond, CA 94804) was spread onto larger 2′ × 4′ trays and left alone for 3 days under ambient conditions. Then, the compost was sieved to 1.2 mm, bagged in a Ziplock with plenty headspace, and acclimatized for 24 h under ambient conditions. Aliquots of the sieved compost (*n* = 3, 5.0 g) were placed in an oven (105 °C, 6 h) fort gravimetric determination of the moisture content, which showed 16.54% moisture (% mc).

Samples of PCL (*n* = 2) and LN-PCL (DP 99 and 35) (*n* = 2) were dissolved in dichloromethane (0.5 g/5 mL) for 9 h under constant mixing with a stir bar (500 rpm). The polymer solution was mixed with 10.0 g of sand (silicon dioxide, washed and dried, Spectrum Chemical, New Brunswick, NJ 08901) using a mortar and pestle, then dried sequentially under ambient conditions in a hood (5 h), then under vacuum (0.5 h). The dried, polymer-coated sand mixture (approximately 10.5 g) was then ground with the pestle before adding 22.0 g of compost in respirometry bottles (1.0 L). Similarly, 0.5 g of LN (*n* = 2) and ground LN-*g*-PCL DP 35 (*n* = 2) were added to respirometry bottles containing 10.0 g of sand and 22.0 g of compost. The baseline (*n* = 2) respirometry bottles contained only sand and compost. An additional 21.5 g of water was added to each of the ten bottles to give a final 57.7% mc compost.

The mineralization of the polymer samples by microorganisms in the compost was studied for 40 days at 25 °C in a respirometer (Columbus Instruments, 3% CO_2_ Sensor) measuring the cumulative carbon dioxide (μL) produced in real-time. Assuming aerobic respiration (carbon source + O_2_ → CO_2_ + H_2_O), the volume of 1 mol of gas at standard temperature and pressure (22.41 L/mol), the atomic mass of carbon (12.0 g/mol), and that each sample was approximately 57% carbon (0.285 g carbon), the potential quantitative conversion of that carbon-to-carbon dioxide was estimated using the following conversion.

2 $$yield\;of\;{CO}_{2}=\frac{carbon\;in\;sample,\;g}{atomic\;mass,\;g/mol}\times \frac{22.4L}{mol}\;gas\;at\;STP$$

This calculated value (μL) allowed a direct determination of percent mineralization taken every 24 h using the cumulative CO_2_ (μL) or by the following equation.

3 $$percent\;mineralization\;\left(\%\right)=\frac{sample{\;CO}_{2\;}\left(\mu L\right)-baseline{\;CO}_{2}\;\left(\mu L\right)}{equation\;2\;value}\times 100\%$$

In addition, each chamber was mixed and the 57% mc maintained by adding water, if needed, twice weekly.

### Agrochemical Release Study

The herbicide release from PCL films was performed by measuring the concentration of MTZ and ATZ released into a phosphate-buffered saline solution (PBS, pH: 7.2) with high-performance liquid chromatography (HPLC, Agilent 1200 Series, CA, USA). Briefly, squares of 2 × 2 cm^2^ were cut randomly from the films, weighed, and placed in 40 mL of PBS solution; the sample container was placed inside a 30 °C incubator shaker (C25KC New Brunswick Scientific, USA) at 100 rpm. 1 mL of the sample solution was taken every week for 10 weeks and replaced with 1 mL of stock PBS solution. To measure the concentrations of metribuzin and atrazine in the sample, 250 μL of sample solution was mixed with 250 μL of acetonitrile over 5 h. The solution was then filtered using a 0.22 μm syringe filter and quantified using HPLC analysis using absorbance measurements at wavelengths of 293 nm for metribuzin and 220 nm for atrazine. For quantification, 20 μL of the sample was injected into an HPLC system equipped with an LC-20AT pump, an SPD-20A PDA detector, a Zorbax Agilent C18 column (150 mm × 4.6 mm inner diameter × 5 μm particle size), and an SIL-20AC autosampler interfaced with the LC- solution software system (Agilent, USA). An elution gradient was used by using water as solvent A and acetonitrile as solvent B. The initial condition was set to 20% of solvent B for 10 min; subsequently, a solvent gradient was set to 90% of solvent B within 10 min and held for 5 min. A flow rate of 1 mL min^–1^ was used for all measurements. The total MTZ and ATZ content in LN-PCL/PCL and PCL films was quantified by immersing a 4 × 4 cm film sample of known weight in 40 mL of acetonitrile (ACN) for 48 h, followed by analysis using HPLC. Recovery results are reported as percentage recovery (% recovery) with relative standard deviation (% RSD) in Table 3.

### Statistical Analysis

Each experiment was replicated three times (*n* = 3), and statistical analysis of the data was performed using RStudio for Windows v2023.06.0 + 421 (RStudio Inc., Boston, MA). Statistical differences were found using one-way ANOVA with a significance level (α) of 0.05; data are reported as means ± standard deviation.

## Results and Discussion

### Chemical Characterization of Composite Films

FT-IR spectra of LN-PCL-containing films (Figure 1) showed peaks characteristic of PCL and LN. PCL-specific peaks were more intense because commercial PCL in which LN-PCL were blended constitutes two-thirds of the film matrix (w/w), shifting the average intensity of the peaks closer to those found in pure PCL films. The functional group peaks observed in PCL are methylene (CH_2_, 2945–2864 cm^–1^), carbonyl (C=O, ∼ 1721 cm^–1^), and C–O/C–C bonds from the PCL amorphous phase (1160–1190 cm^–1^).^33^ Weak hydroxyl (OH, 3000–3700 cm^–1^) peaks typical of LN, observed in pure LN spectra (Figure S1), are not discernible here due to their low intensity and overlap with the dominant PCL matrix. This low intensity is expected given the high PCL content, which masks LN’s OH contribution.

**Figure 1:**
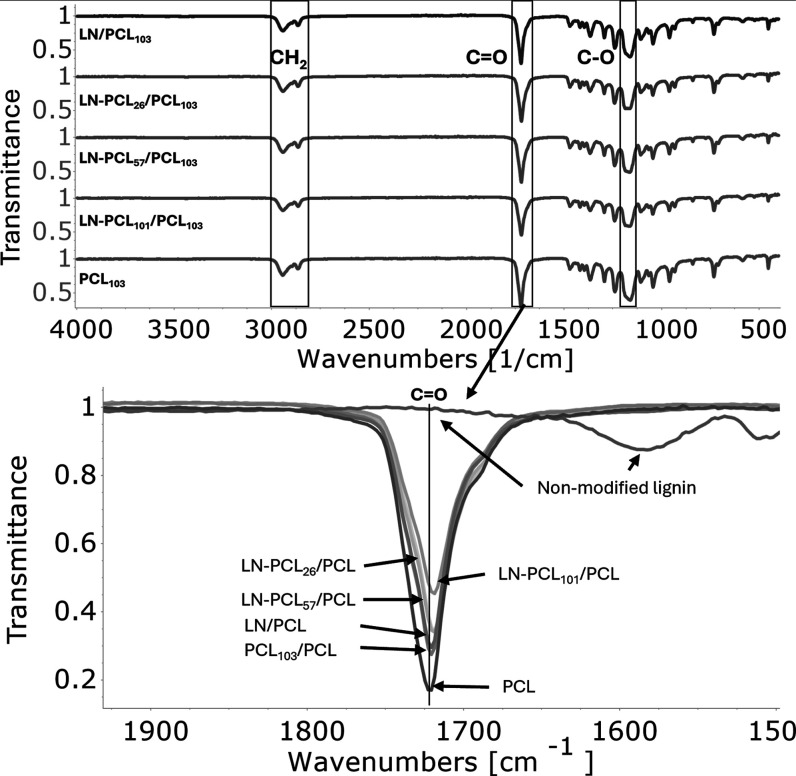
FT-IR spectra of PCL films that contain four different types of filler: LN and LN-PCL with three different DPs (26, 57, and 101). The spectrum of PCL_103_ is shown for comparison.

In a study published on LNPs blended as a filler in chitosan/poly vinyl alcohol (CS/PVA) coating films, hydrogen bond interactions between LNPs and CH/PVA blends were indicated by a shift of C–O, from 1073 cm^–1^ to 1081 cm^–1^, after the incorporation of 1 to 5% LNPs.^25^ Similarly, we observed a slight C=O peak shift from 1720 cm^–1^ to 1719 cm^–1^ after incorporating LN-PCL polymers (Figure 1), suggesting hydrogen bonding between LN’s OH and PCL’s C=O groups. The sharp C–O–C peak at 1166 cm^–1^ also shifted to 1186 cm^–1^ in LN-PCL_26_/PCL and broadened in other samples (Figure S2), indicating overlap between PCL’s C–O–C and LN’s C–O groups. This shift, more pronounced in LN-PCL_26_/PCL, may result from shorter PCL chains exposing more LN OH groups and stiffening C–O–C vibrations. Longer PCL chains (e.g., LN-PCL_101_) may restrict LN-PCL interactions, limiting access to LN’s functional groups.^34^ In contrast, FT-IR of PCL with nonmodified LN showed no shifts compared to pure PCL, suggesting primarily physical interactions.^33^

### Chemical Characterization of PCL Films Loaded with Herbicides

FT-IR spectra of PCL films loaded with metribuzin (MTZ) and atrazine (ATZ) are shown in Figures 2 and 3. For MTZ-loaded films, characteristic herbicide peaks include weak symmetric and asymmetric amine (−NH_2_) stretches at 3313 cm^–1^ and 3196 cm^–1^ (Figure S3), carbonyl amide (C­(=O)­N) at 1684 cm^–1^, and imine (C=N) at 1624 cm^–1^ (Figure S4). For ATZ-loaded films, distinct peaks include the amino group (N–H) at 3254 cm^–1^ (Figure S5), the triazine group at 1546 cm^–1^ (Figure S6), and the triazine ring sextant at 805 cm^–1^.

**Figure 2:**
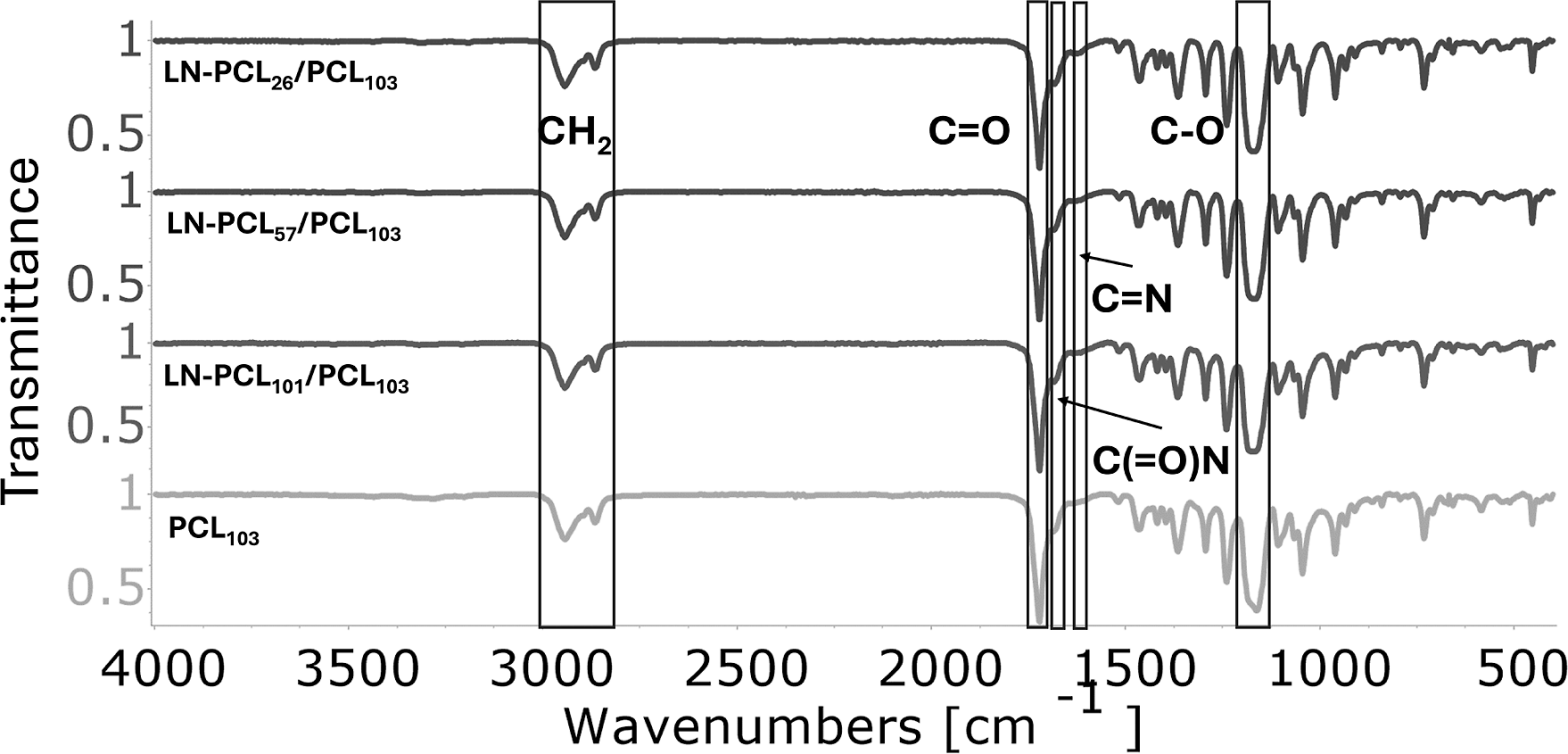
FT-IR spectra of PCL films loaded Metribuzin that contain three different types of filler: LN-PCL with three different DPs (26, 57, and 101). The spectrum of PCL_103_ is shown for comparison.

**Figure 3:**
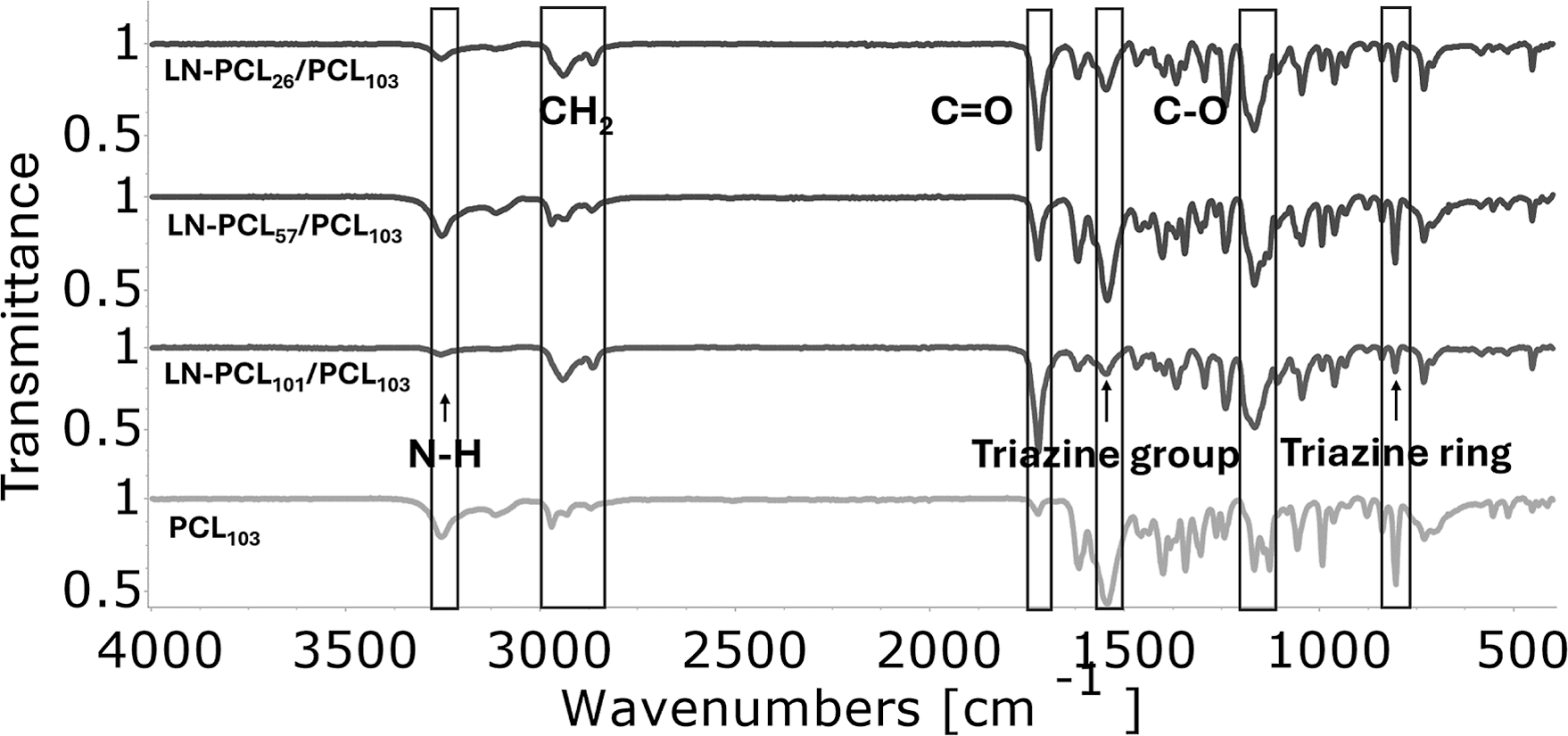
FT-IR spectra of PCL films loaded Atrazine that contain three different types of filler: LN-PCL with three different DPs (26, 57, and 101). The spectrum of PCL_103_ is shown for comparison.

Incorporation of MTZ broadens the –NH peak to 3280 cm^–1^, suggesting weak hydrogen bonding with PCL’s C=O. For ATZ-loaded films, the N–H peak at 3254 cm^–1^ varies in intensity: high in LN-PCL_57_/PCL and pure PCL films, indicating stronger interactions with PCL’s C=O or LN’s –OH, and lower in LN-PCL_26_/PCL and LN-PCL_101_/PCL, reflecting weaker interactions due to poor dispersion. Short chains in LN-PCL_26_/PCL may limit mixing, while long, tangled chains in LN-PCL_101_/PCL may shield the functional groups. The absence of peak shifts suggests physical dispersion effects rather than chemical bonding. Additional shifts include C–H stretching from 2944 cm^–1^ to 2971 cm^–1^ in LN-PCL_57_/PCL and PCL films, indicating altered CH_2_ environments, and a reduced C=O peak intensity at 1722 cm^–1^, correlating with ATZ dispersion. The C–O–C peak at 1166 cm^–1^ shifts to 1128 cm^–1^ in LN-PCL_57_/PCL and PCL films (Figure S7), possibly due to hydrogen bonding with ATZ, enhancing integration.

The ^1^H NMR analysis of the LN-PCL/PCL also identified peaks attributed to PCL chains (Figure 4), including: repeating –CH_2_O- (4.03 ppm, d), terminal –CH_2_OH (3.65 ppm, d’), –COCH_2_– (2.29 ppm, a), –CH_2_–, (1.63 ppm, b), and –CH_2_– (1.38 ppm, c).^10,30^

**Figure 4:**
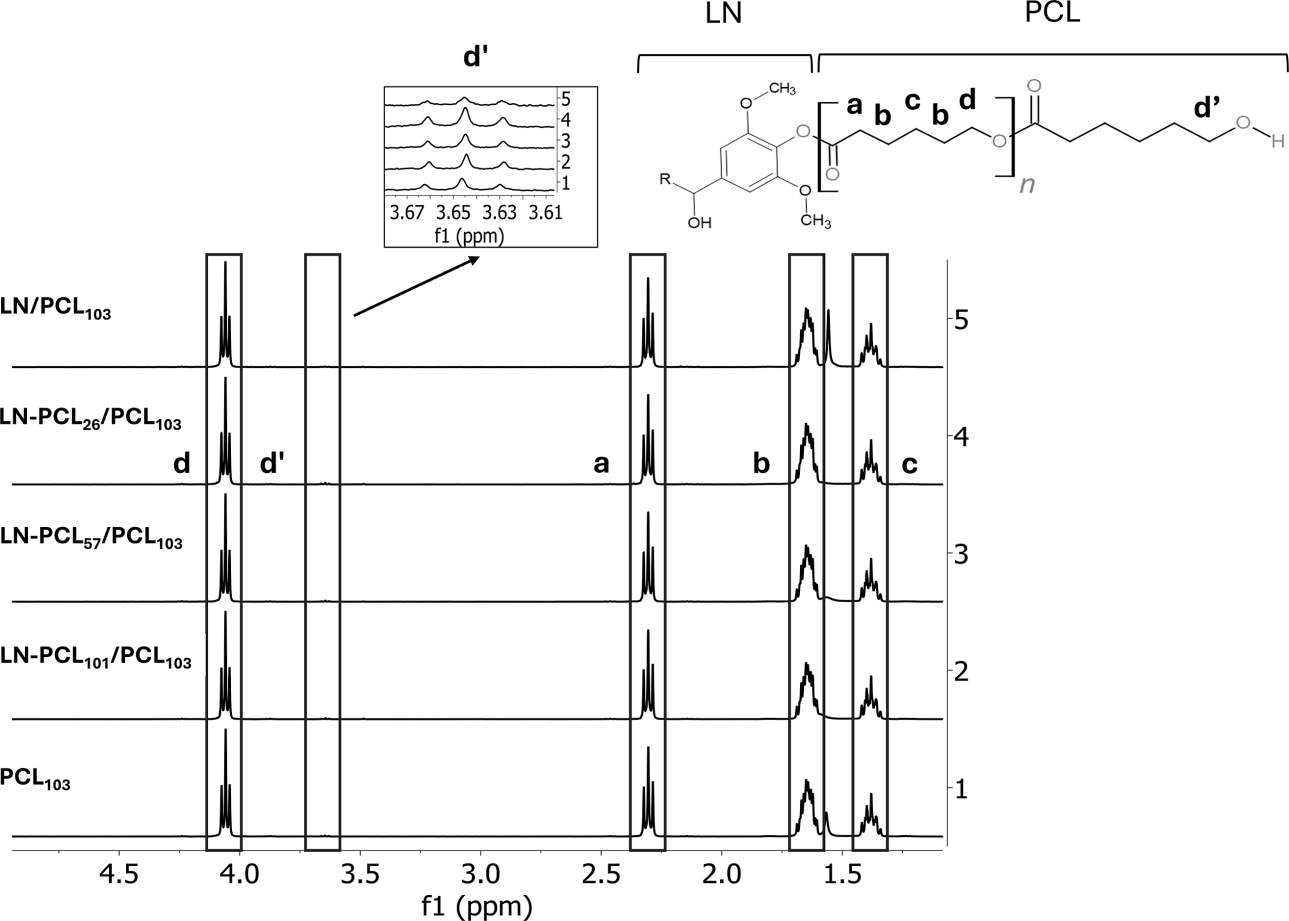
^1^H NMR spectra for PCL films that contain four different types of filler: LN and LN-PCL with three different DPs (26, 57, and 101). The spectrum of PCL_103_ is shown for comparison.

The blending process of PCL with LN-PCL_57_ and LN-PCL_101_ results in no reduction in the DP of PCL as calculated based on the NMR data, indicating a physical mixture of the polymers rather than cross-linking between them. Similarly, the reintroduction of −CH_2_OH peaks after the incorporation of LN into a PCL matrix indicates the absence of cross-linking during film formation. On the other hand, the incorporation of LN-PCL_26_ polymer resulted in a reduction in DP. These observations suggest a possible cross-linking between methylene (−CH_2_O−) in the repeated units of PCL and LN-PCL polymers with low DP. The cross-linking was also noted by a hydrogen bond formation on FTIR analysis suggesting that a short length of the PCL chains may allow for a more intense interaction between PCL_103_ and the functional groups of LN by hydrogen bonds.

### Thermal and Physical Characterization of Films

Thermal analysis using DSC showed no differences in melting temperature (*T*_m_) across samples compared to the *T*_m_ of pure PCL (Table 1). However, the crystallization temperature (*T*_c_) was slightly higher for PCL films containing LN or LN-PCL (Table 1). In contrast, a study using brewer’s spent grain (BSG) as a lignocellulose filler reported no effect on *T*_m_ and a slightly lower *T*_c_ for the PCL matrix, attributed to restricted PCL chain mobility.^35^

**Table 1:** Thermal Properties of LN-PCL/PCL Composite Films^a^

sample	*T*_c_ (°C)	Δ*H*_c_ (J/g)	*T*_m_ (°C)	Δ*H*_m_ (J/g)	*X*_c_ (%)
LN/PCL	29.4	46.4	54.4	46.1	33.9
LN-PCL_26_/PCL	30.3	68.9	54.5	68.8	50.6
LN-PCL_57_/PCL	30.6	59.1	55.3	58.4	42.9
LN-PCL_101_/PCL	31.7	68.2	55.9	66.6	49.0
PCL_103_	28.8	54.8	55.2	54.4	39.9

a *T*_c_: crystallization temperature, Δ*H*: Enthalpy, *T*_m_: melting temperature, and *X*_c_: crystallization %.

DSC results revealed that the percent crystallization (*X*_c_), enthalpy of crystallization (Δ*H*_c_), and enthalpy of melting (Δ*H*_m_) varied with the filler type (Table 1). LN/PCL films exhibited lower *X*_c_, Δ*H*_c_, and Δ*H*_m_ compared to pure PCL, reflecting an amorphous phase introduced by LN that restricts PCL chain movement and crystallization.^35^ By incorporation of an amorphous phase like LN, the movement of PCL is limited to form crystallization. In contrast, the incorporation of LN-PCL resulted in films with higher *X*_c_, Δ*H*_c_, and Δ*H*_m_ values, indicating a more ordered PCL chains arrangement. For instance, LN-PCL_101_/PCL (DP ∼ 101) had higher *X*_c_ than PCL_103_ (DP ∼ 103), despite similar DP, suggesting that grafted LN-PCL enhances chain alignment and promotes crystallization.

Similar results were found in a previous study evaluating the *X*_c_ of LN-PCL polymers,^10^ where results showed higher *X*_c_ at lower DP (e.g., 50.6 ± 3.3% at DP 22 vs 49.0 ± 2.2% at DP 101) due to greater chain mobility at low DP. Here, LN-PCL’s short chains (e.g., LN-PCL_26_) facilitate diffusive motion, aiding crystal formation, while longer chains (e.g., LN-PCL_101_) limit mobility, yet still yield higher *X*_c_ than neat PCL_103_, likely by reducing amorphous phases formed during solvent evaporation.

TGA analysis of films with and without LN or LN-PCL polymers revealed enhanced thermal stability in LN- or LN-PCL-containing films (Figure 5). LN/PCL films showed better stability above 400 °C but a lower maximum rate of degradation temperature (MRDT) of 333.72 °C compared to 353.64 °C for pure PCL. A similar trend was observed in films containing LN-PCL polymer with MRDT values of 353.64 °C (DP 101) and 345.94 °C (DP 26). These results align with BSG-filled PCL composites, which exhibited lower mass loss above 400 °C and reduced MRDT.^35^ Another study reported increased thermal stability (half-weight loss temperature rising from 346.51 to 364.11 °C) with 1–5% LNPs in CS/PVA blends.^25^ The lower MRDT in LN-containing films may stem from LN’s functional groups acting as radical initiators, accelerating PCL ester bond hydrolysis under thermal stress. TGA hence corroborates the physical rather than chemical incorporation of LN into the PCL matrix.

**Figure 5:**
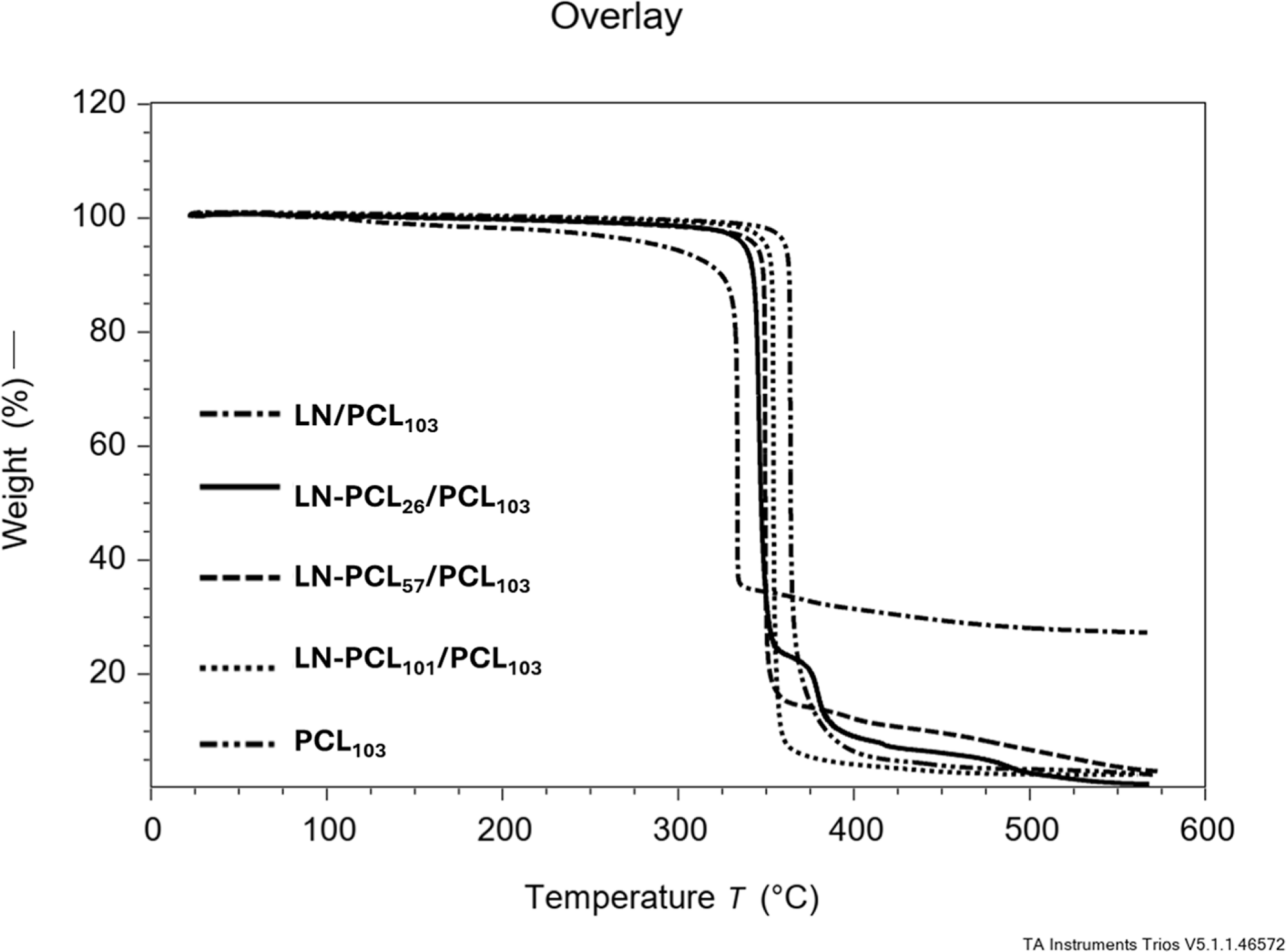
TGA analysis for PCL films prepared with different LN-PCL fillers (26, 57, and 101 DP).

Additionally, TGA results revealed a high mass residue (∼35%) at 600 °C for LN/PCL_103_, unlike other samples, which approach a near-zero residue. This elevated residue reflects the ∼ 33% (w/w) LN content in LN/PCL_103_, comparable to the LN proportion in LN-PCL/PCL films. In LN-PCL polymers (DP > 20), PCL dominates, leading to a decomposition profile similar to neat PCL, with minimal residue. However, in LN/PCL_103_, the higher LN fraction accelerates initial PCL decomposition via functional groups acting as hydrolysis initiators at elevated temperatures, leaving substantial residue due to LN’s thermal stability.

The mechanical characteristics of PCL film composites obtained from stress–strain curves are shown in Figures 6 and 7. Pure PCL films presented a tensile strength of 17.42 ± 3.73 MPa and an elastic modulus of 0.32 ± 0.05 GPa. However, these PCL films exhibited a low elongation at break of (8.28 ± 3.73%), indicating a tendency toward brittleness. Previous literature has shown that PCL is characterized by having average tensile strength between 20 and 30 MPa and more than 100% in elongation at break; differences in elongation at break could be related to different *M*_w_ (>80, 000 g/mol) and methodologies (extrusion or hot press plates).^35–37^

**Figure 6:**
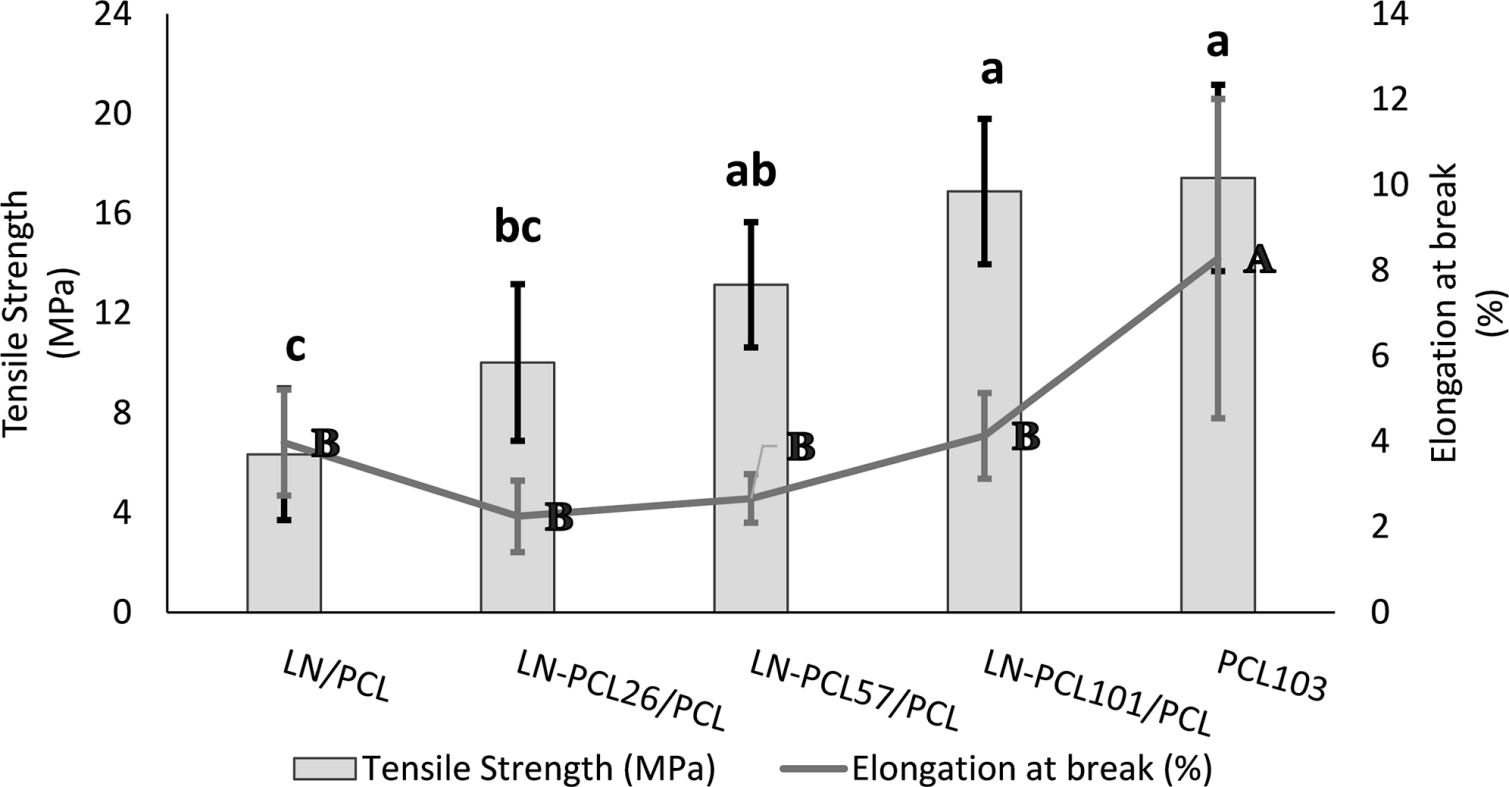
Tensile strength (MPa) and elongation at break (%) for PCL films prepared with different LN-PCL fillers (26, 57, and 101 DP). Means ± SD with different lowercase letters (a–d) above bars indicate statistically significant tensile strength differences (*p* < 0.05) between means for different treatments. Means ± SD with different uppercase letters (A–D) indicate significant elongation at break differences (*p* < 0.05) between means for different treatments.

**Figure 7:**
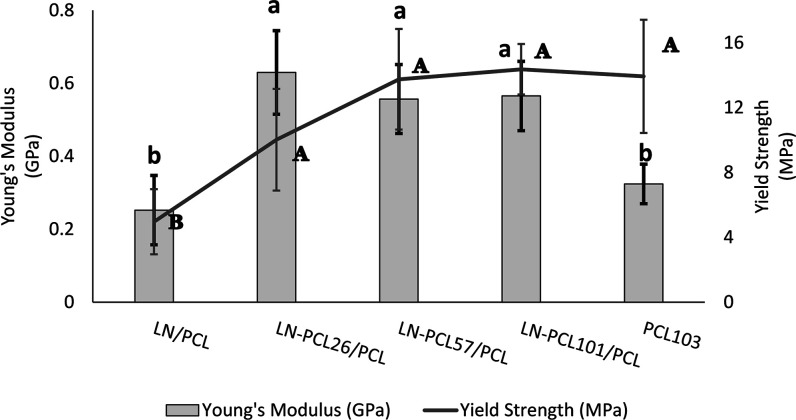
Young’s modulus (GPa) and yield strength (MPa) for PCL films prepared with different LN-PCL fillers (26, 57, and 101 DP). Means ± SD with different lowercase letters (a–d) indicate significant differences (*p* < 0.05) in Young’s modulus between treatments. Means ± SD with different uppercase letters (A–D) indicate significant differences (*p* < 0.05) in yield strength between means for different treatments.

The addition of LN to the PCL matrix decreased tensile strength by ∼ 3 fold compared to pure PCL film, and the elongation at break also was reduced by ∼ 50% relative to the PCL films with no filler added. While no differences were found in elongation at break between LN-containing films and LN-PCL-containing films, we found that an increase in DP of PCL in LN-PCL polymers caused an increase in tensile strength from 10.02 ± 3.14 MPa (for LN-PCL_26_) to 16.88 ± 2.91 MPa (LN-PCL_101_), possibly due to enhanced interfacial interaction between the polymers. The higher elongation at break of LN-PCL_101_ was similar to that of the pure PCL film with no additives. The lower tensile strength and elongation in the polymeric films containing LN can be explained by the weak compatibility between the two polymers, possibly due to the limited mobility of PCL chains and the amorphous structure of LN.^33,35^ Similar behavior was found in a previous study of PLA bionanocomposites containing LNPs,^11^ in which the solvent casting method was used. With this synthesis method, a decreased tensile strength was observed due to the inhomogeneous dispersion of LNPs in the solvent-casting film.

The addition of LN-PCL to PCL films however resulted in a 2-fold increase in Young’s modulus regardless of DP. The enhancement in stiffness of the material is related to the ability of the film to return to its original form after being exposed to a force. This increase in modulus may be related to the limited mobility of PCL chains and the possible hydrogen bond formation between the carbonyl group of PCL and functional groups of LN^33^ which is consistent with our findings in the chemical characterization of PCL films.

### Surface Characterization of Films

The surface wettability of both the PCL and LN-PCL polymer films, as determined by contact angle measurement, was in the hydrophobic range. For both faces of the films, the observed CA ranged between 60° and 90° across all film types (Figures 8 and 9), consistent with previous literature on PCL-containing films. This reflects the hydrophobic nature of PCL, which exhibits a low affinity for polar substances such as water.^38^

**Figure 8:**
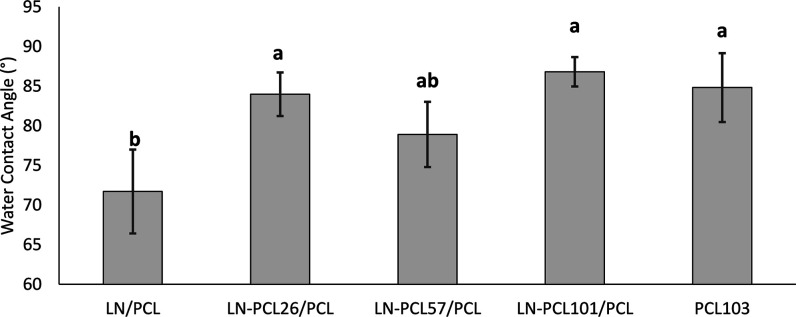
Water contact angle measurements for upper faces of PCL films prepared with different LN-PCL fillers (26, 57, and 101 DP). Different letters (a–c) indicate significant differences between means ± sd. Error bars = SD.

**Figure 9:**
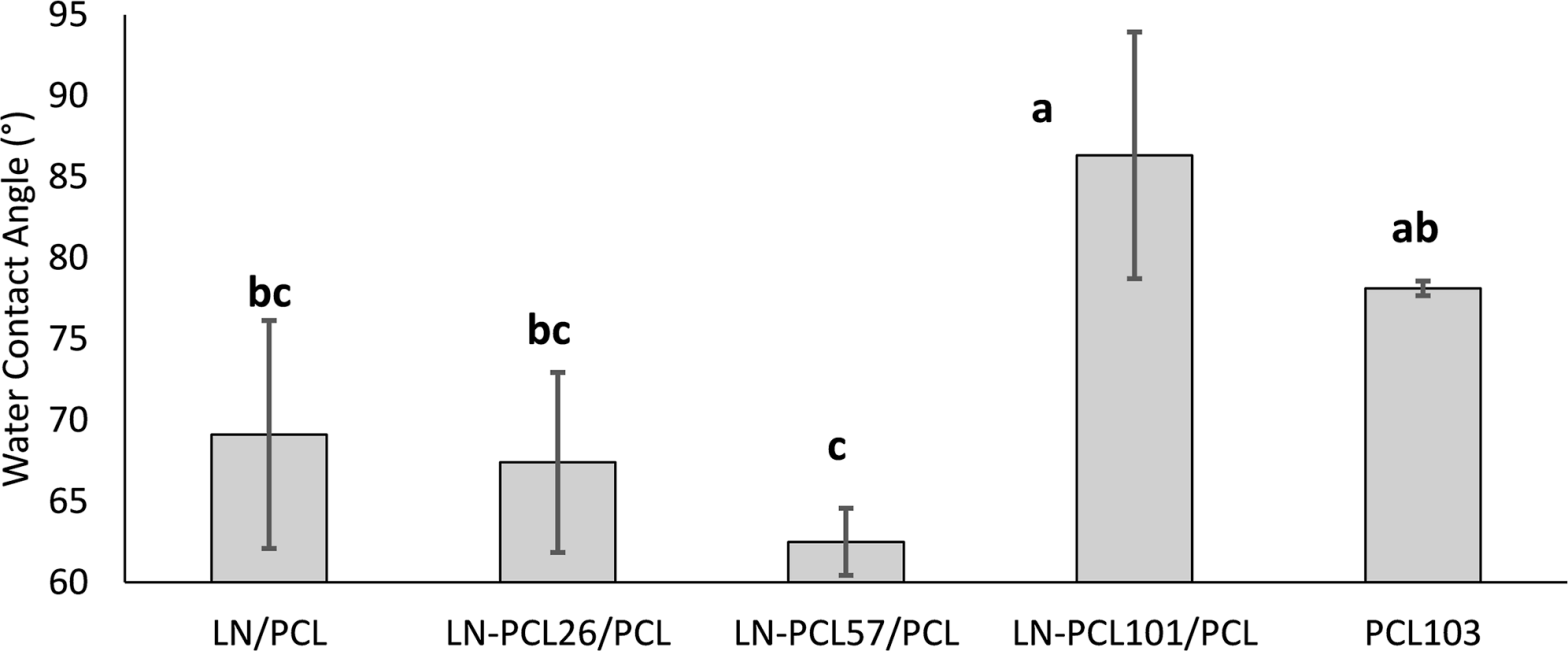
Water contact angle test on bottom sections for PCL films prepared with different LN-PCL fillers (26, 57, and 101 DP). Different letters (a–c) indicate significant differences between means ± sd.

Interestingly, we observed differences in CA between the top and bottom faces of the films (“bottom” refers to the side of the film facing the PTFE container at casting and “top” is the side facing the air). PCL films containing LN presented lower CAs on both sides compared to CAs on PCL_103_ films. While the top side generally exhibited higher CAs, no significant differences were associated with DP for LN-PCL composites. On the other hand, the bottom sides of the films showed significant changes in CA values due to variations in DP of PCL in the LN-PCL-containing films. A low DP in LN-PCL_26_ and LN-PCL_57_ resulted in lower CA values at the bottom of the films. However, the high DP in LN-PCL_101_ resulted in no significant differences in CA values compared to the pure PCL_101_ film. The differences in CA between the top and bottom sides of films can be explained by the irregularities resulting from the interaction between polar functional groups of LN and the hydrophobic surface of the PTFE dish (CA ∼ 103°), leading to the formation of holes.

SEM was used to examine the surface topography of the films made with different polymer blends (Figures 10 and 11). SEM images revealed textural differences between the bottom and top surfaces of the films. The top faces of the films presented differences across the different polymer blends (Figure 10). Pure PCL films presented an organized surface with visible pores formed during the evaporation of the organic solvent, which are not present, especially at a higher DP of the grafted polymer. The bottom surfaces were smooth for the LN-PCL_101_/PCL composite and PCL control films (Figure 11). The LN and LN-PCL containing films presented holes possibly caused by the repulsion between the polar functional groups of LN and the hydrophobic surface of the PTFE dish.

**Figure 10:**
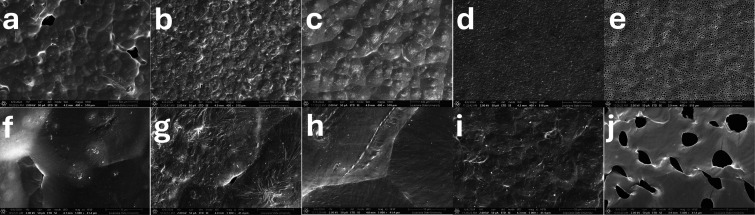
SEM images of top faces of films made with (a,f) LN/PCL, (b,g) LN-PCL_26_/PCL, (c,h) LN-PCL_57_/PCL, (d,i) LN-PCL_101_/PCL, and (e,j) PCL_103_. Magnification of ×400 was used for a–e and ×5000 for f–j.

**Figure 11:**
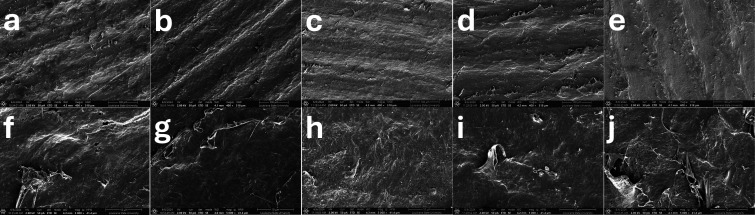
SEM images of bottom faces of films made with (a,f) LN/PCL, (b,g) LN-PCL_26_/PCL, (c,h) LN-PCL_57_/PCL, (d,i) LN-PCL_101_/PCL, and (e,j) PCL_103_. Magnification of ×400 was used for a–e and ×5000 for f–j.

### Degradation Analysis of Polymers

The degradability of LN, PCL, and LN-PCL polymeric materials in compost at 25 °C was studied by using respirometry over a 40 day period. From eq 2, quantitative (complete) conversion of the 0.5 g of each sample with approximately ∼ 63% carbon content would yield a maximum of ∼ 600,000 μL of carbon dioxide (CO_2_) (Table 2). The production of CO_2_ was indicative of aerobic cellular respiration by microorganisms in the organic compost, which began almost immediately (Figure 12). The cumulative CO_2_ (μL) captured in the bottles was measured and recorded every 120 min, though the data was plotted every 24 h. At around 30 days of the experiment, the cumulative CO_2_ reached steady state, suggesting the fullest extent of degradation, and so the experiment was terminated at 40 days. While PCL degraded the most in compost, reaching 202,708 μL of CO2, or 34.4% mineralization at 40 days using eq 3, the maximum amount of CO_2_ produced from LN was 8965 μL on day 3 of the experiment, or just 1.48% mineralization (Table 2, Figure 13). The LN curve showed a negative trend with respect to the baseline, suggesting a deleterious effect on the compost microbiome attributable to the accumulation of degradation products from LN. The PCL, LN-PCL_35_, and LN-PCL_99_ samples all showed increasing degradation in the first 30 days before reaching steady-state. PCL had the highest degradation, while the LN-PCL_35_ and LN-PCL_99_ showed lower degradation, with a direct relationship between degradation and the degree of polymerization of PCL grafted to LN. However, the increase in degradation percentages and the increase in wt % of PCL polymerized on LN at DP 35 and 99 was not a 1:1 translation (Table 2), i.e., the mechanism of how polymerization improved degradation is inconclusive. Moreover, given that the mineralization of these materials reached steady state at 25 °C in 40 days suggests that significantly higher degradation of these materials would be achieved at higher temperatures, such as 58.5 °C or industrial compost temperatures necessary to activate thermophilic microorganisms.

**Table 2:** Aerobic Respirometry of 0.5 g of Carbon-Containing Sample at 25 °C and 57% Moisture Content Showing Cumulative Carbon Dioxide Production and Mineralization after 40 Days

samples	weight percent carbon (w %)	quantitative conversion (μL)	cumulative CO_2_ (μL)	mineralization (%)	Av. Coefficient of variance (<5.0)
PCL	63.1	589,196	202,708	34.40	4.77
LN	65.0	606,938	8,965^a^	1.48	1.93
LN-PCL_35_ 70 wt % PCL	63.2	590,130	77,356	13.11	3.44
LN-PCL_99_ 86.8 wt % PCL	63.6	593,865	119,506	20.12	1.70

a Maximum degradation at time = 3 days for LN.

**Figure 12:**
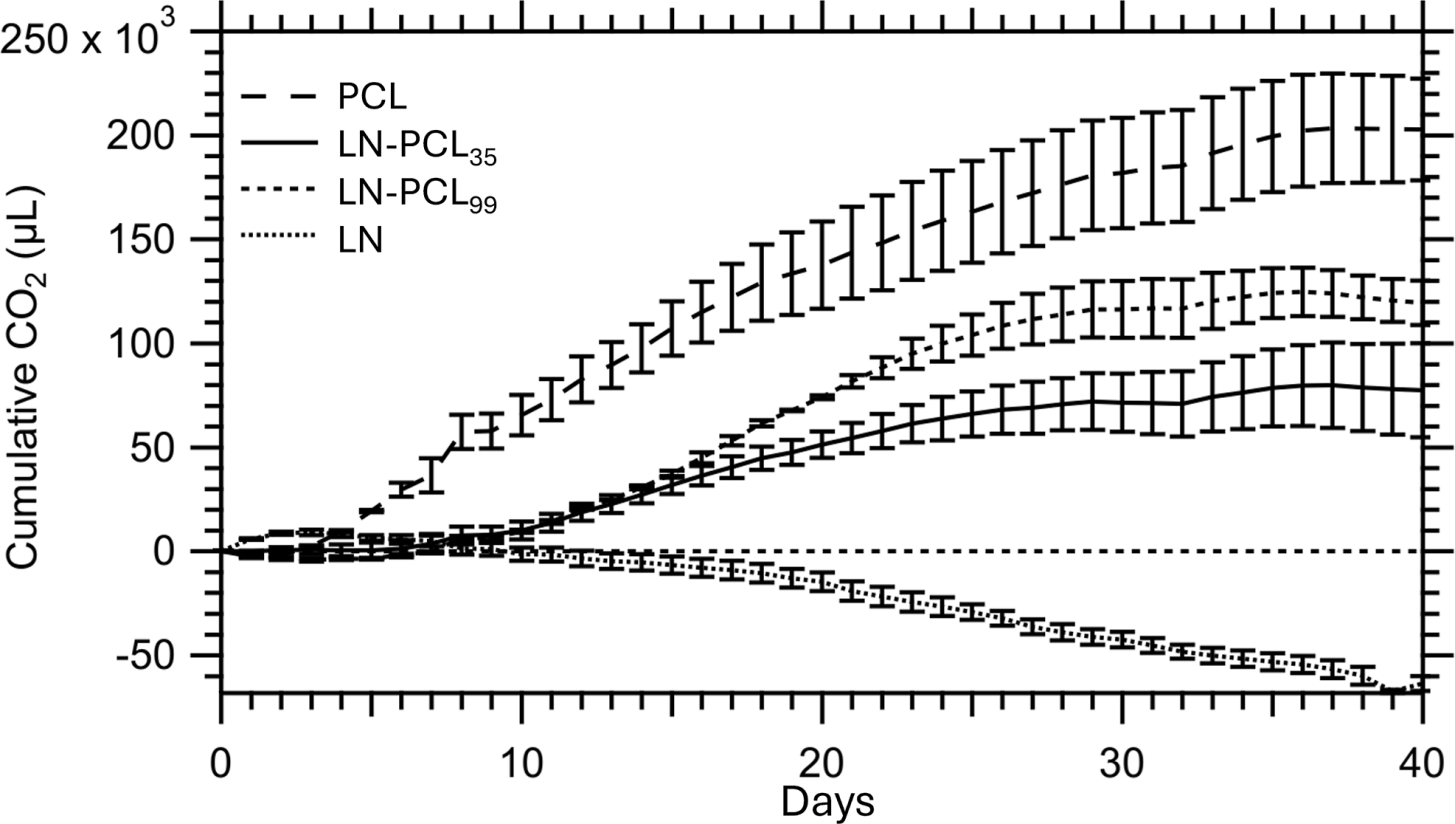
Cumulative carbon dioxide emissions of 0.5 g of carbon-based samples in organic compost under respirometry conditions of 25 °C and 57% moisture content (baseline subtracted).

**Figure 13:**
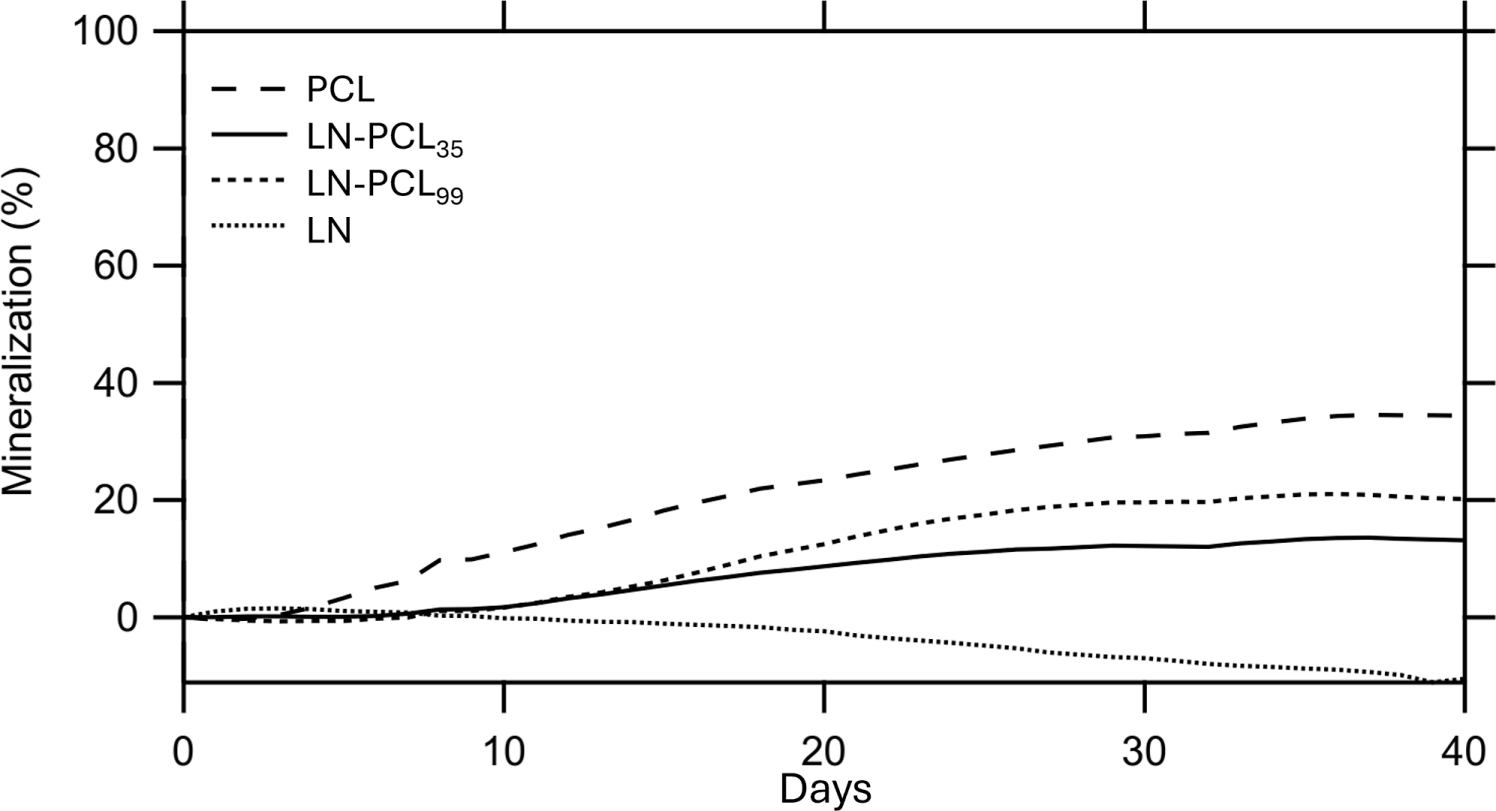
Percent mineralization of 0.5 g of carbon-based samples in organic compost under respirometry conditions of 25 °C and 57% moisture content (baseline subtracted).

### Optical Characterization of Films

Pure PCL films presented a transmittance of UV light of 43.12% for UV-A/B and 45.87% for UV–C (Table 3). This moderate UV light blocking by PCL is expected since it is translucent and colorless. All films containing LN transmitted less UV light compared with pure PCL films. This is attributed to LN’s UV absorbing properties, related to the chromophores within LN’s structure, in particular its phenolic and ketone groups.^39,40^ Similar outcomes were observed with the incorporation of micro- and nanoparticles into various polymeric films, including PLA,^39–42^ PCL/PLA,^37^ poly­(butylene adipate-*co*-terephthalate),^43^ and polycyanoacrylate.^44^ Increased LN concentration has been found to be correlated with improved UV light protection.^39–41^ However, the modification of phenolic groups on LN has demonstrated a reduction in the UV light blocking.^41,43^

**Table 3:** Thickness, UV Light Transmittance (%) of UV-AB (280–400 nm) and UV–C (220–275 nm), and Herbicide Recovery (% Recovery, % RSD) for PCL films Prepared with Different LN-PCL Fillers (26, 57, and 101 DP). Means ± SD with Different Letters (a–d) Indicate Significant Differences between Treatments Means *I* = 3)

sample	thickness (mm)	UVAB (280–400 nm) %	UVC (220–275 nm) %	MTZ % recovery (% RSD)	ATZ % recovery (% RSD)
LN/PCL	0.09 ± 0.00^b^	99.14 ± 0.00^a^	98.17 ± 0.00^a^		
LN-PCL26/PCL	0.09 ± 0.01^b^	99.37 ± 0.02^a^	94.50 ± 0.00^b^	93(0.4)	109(0.07)
LN-PCL57/PCL	0.09 ± 0.01^b^	97.99 ± 0.09^b^	92.05 ± 1.06^c^	104(2)	113(0.65)
LN-PCL101/PCL	0.09 ± 0.01^b^	99.15 ± 0.07^a^	95.72 ± 1.06^b^	102(11.1)	112(0.53)
PCL103	0.11 ± 0.01^a^	56.88 ± 0.36^c^	54.13 ± 0.00^d^	103(0.1)	105(0.29)

PCL films with LN-PCL blocked significantly more UV light compared to pure PCL films, with transmittance ranging from 2.01% to 0.63% for UV-A/B and 7.9% 5 to 4.28% for UV–C. Although small differences were found in UV transmittance with varying DP of the incorporated LN-PCL, no clear trend was observed.

Chemical modification of LN through PCL grafting by ROP in this study did not significantly affect the UV transmittance of films, unlike modification with citric acid (18%) and acetylation (14%).^41^ These results suggest homogeneous LN modification between polymers, preserving phenolic and ketone groups, as found by others.^40^ UV chromophores, which are responsible for UV light absorption, are formed during polymerization at LN coupling sites, potentially explaining the lack of reduction in UV light blocking by the films containing LN-PCL.^40^

### Agrochemical Release Study

After an initial burst release on the first day, the hydrophilic herbicide MTZ was released at an average rate between 37 and 42% for all films over 70 days (Figure 14a). This rapid initial release was anticipated given the high solubility of MTZ in water (1050 mg/L). This rapid initial release was attributed to the swelling and relaxation of PCL in water at pH 7, where water molecules diffuse through the amorphous phases of the polymeric matrix and interact with PCL’s hydrophilic carbonyl groups via hydrogen bonding.^45^ This swelling facilitates the rapid diffusion of MTZ near the film surface. A previous study reported that ATZ release from PCL nanoparticles in water was similarly controlled by polymer chain relaxation, with an initial burst of ∼ 20% followed by a slower release after 2 days.^46^ In comparison, our ATZ-loaded films exhibited a lower initial burst (9:13%, Figure 14b) over 70 days, likely due to the denser film matrix compared to nanoparticles, which restricts diffusion.

**Figure 14:**
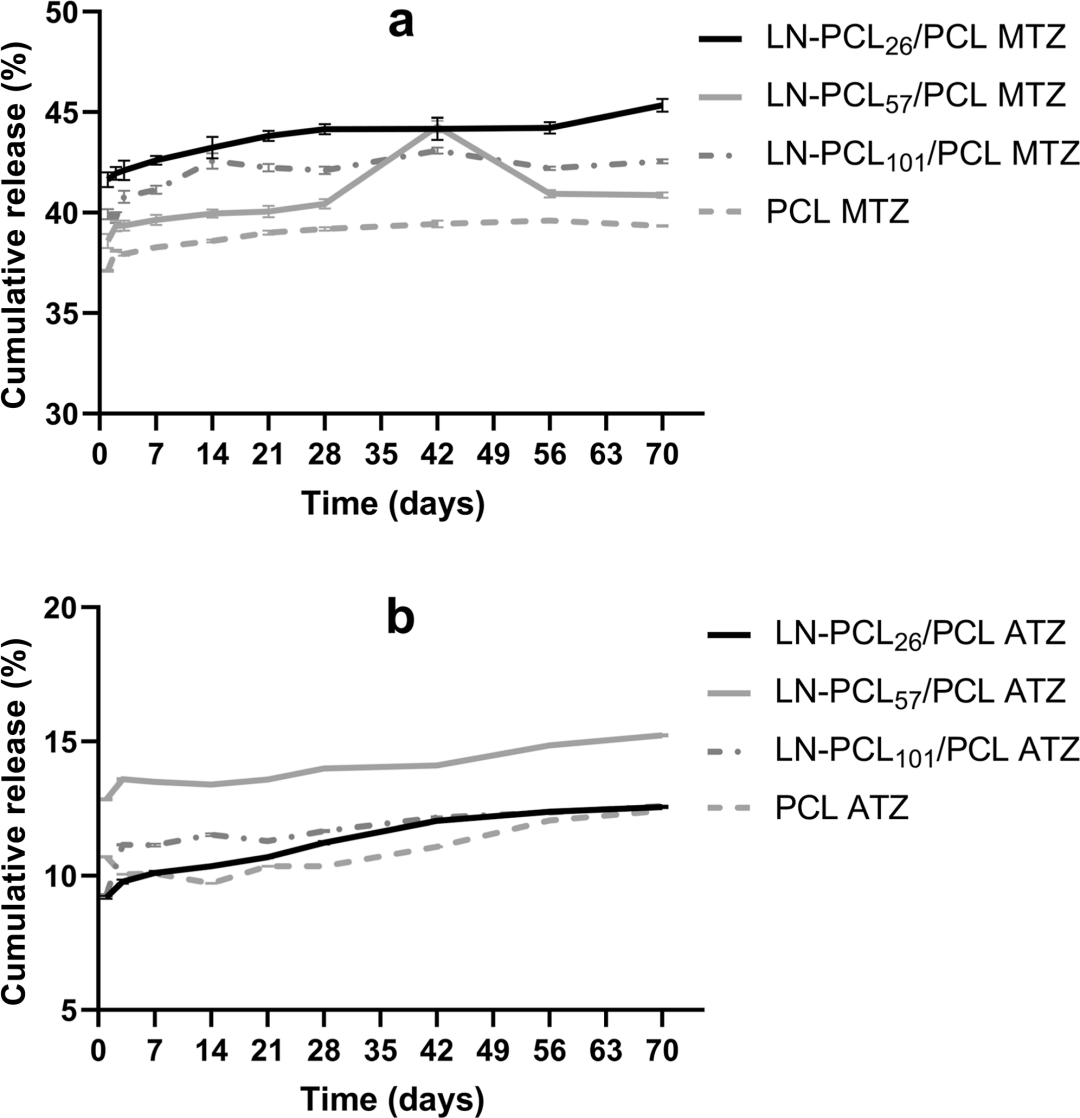
Release profile of MTZ (a) and ATZ (b) at 30 °C for PCL films prepared with different LN-PCL fillers (26, 57, and 101 DP) (*n* = 3).

Films released MTZ differently depending on their composition in this order: LN-PCL_26_ > LN-PCL_101_ > LN-PCL_57_ > PCL. The enhanced release from LN-PCL-containing films relative to pure PCL can be attributed to the hydrophilicity of LN in the grafted polymer. This hydrophilicity likely increases water molecule interaction with the matrix, thereby enhancing the diffusion of MTZ. Additionally, surface irregularities in LN-PCL_26_-containing films may further facilitate water molecule penetration, increasing MTZ diffusion. While the porosity of the PCL films surface contributes to the initial burst, FT-IR analysis indicates weak hydrogen bonding between MTZ’s –NH groups and PCL’s C=O (broadened –NH peak at 3280 cm^–1^), which may slow MTZ diffusion after the burst by anchoring it within the matrix, particularly in pure PCL films, resulting in a slower release.

Conversely, the burst release of the more hydrophobic herbicide ATZ was lower (9–13% for all films; Figure 14b). Further analysis over a 70 day period revealed that ATZ released gradually, with pure PCL_103_ films showing the slowest rate, which could be attributed to the compatible hydrophobic nature of ATZ and PCL. FT-IR results reveal stronger hydrogen bonding in LN-PCL_57_/PCL and PCL films (high N–H peak intensity at 3254 cm^–1^), enhancing ATZ dispersion and slowing release, while weaker interactions in LN-PCL_26_/PCL (lower intensity) correlate with slightly faster release due to poorer integration.

The results suggest a diffusion-controlled release process in two phases: an initial fast release from the film surface, followed by a slower release as the herbicide diffuses from within the film due to PCL chains relaxation. It is important to note that herbicide release was measured in PBS and may differ in soil. Degradation studies under soil compost conditions suggest that LN in the films might reduce degradation rates, potentially leading to slower release in LN-PCL-containing films, with a higher DP correlating with faster degradation.

In summary, we explore the modification of LN to form LN-PCL with different DP of PCL attached to LN using the ROP method. We focused on evaluating the effects of incorporating LN-PCL with different DPs into PCL films and the influence of different DPs on the physical-chemical properties of these films. The incorporation of LN-PCL into PCL films resulted in possible hydrogen bond formation, as revealed by FTIR and NMR. Thermal properties of the films showed higher crystallinity after the addition of LN-PCL while mechanical analysis revealed an increase in flexibility maintaining the structural integrity of the composite film. Surface evaluation exhibited that lower DPs in LN-PCL copolymers lead to more hydrophilic surfaces with irregularities on films compared to films made of only PCL. LN-PCL-containing films had >94% UV absorbance, much improved over that of PCL films. The films controlled the release of herbicides, with hydrophobic ATZ releasing more slowly than that of hydrophilic MTZ from all films over the 10 weeks of release. Overall, this study demonstrates the potential of incorporating LN-PCL polymers into a PCL matrix to create multifunctional films with enhanced physical properties, providing UV protection and controlled herbicide release for improved efficacy and biodegradability. These films hold promise for future use in sustainable agriculture, horticulture, and weed management systems, where they can reduce the reliance on persistent herbicides and minimize environmental impact. Additionally, the controlled release of herbicides enhances efficacy, enabling lower application rates compared to those of conventional formulations. The use of biodegradable carriers, such as lignin and PCL, also mitigates toxicity to nontarget plants, addressing challenges related to crop safety and soil health.

## Supplementary Material



## Data Availability

Data will be made available from the corresponding author upon request.

## Notes

FT-IR spectra of 3100–3800 cm-1 region for PCL films that contain four different types of fillers: LN and LN-PCL with three different DPs (26, 57, and 101); FT-IR spectra of 1000–1400 cm^–1^ region for PCL films that contain four different types of filler: LN and LN-PCL with three different DPs (26, 57, and 101); FT-IR spectra of 3150–3800 cm^–1^ region for MTZ-loaded PCL films that contain four different types of filler: LN and LN-PCL with three different DPs (26, 57, and 101); FT-IR spectra of 1550–1750 cm^–1^ region for MTZ-loaded PCL films that contain four different types of filler: LN and LN-PCL with three different DPs (26, 57, and 101); FT-IR spectra of 2800–3400 cm^–1^ region for ATZ-loaded PCL films that contain four different types of filler: LN and LN-PCL with three different DPs (26, 57, and 101); FT-IR spectra of 1450–1900 cm^–1^ region for ATZ-loaded PCL films that contain four different types of filler: LN and LN-PCL with three different DPs (26, 57, and 101); and FT-IR spectra of 1050–1300 cm^–1^ region for ATZ-loaded PCL films that contain four different types of filler: LN and LN-PCL with three different DPs (26, 57, and 101) (PDF)

## Abbreviations

ATZ: atrazine
C=O: carbonyl group
CDCl_3_: deuterated chloroform
CH_2_: methylene
CL: ε-caprolactone
DCM: dichloromethane
DP: degree of polymerization
DSC: differential scanning calorimetry
FT-IR: Fourier transform infrared spectroscopy
HPLC: high-performance liquid chromatography
LN: lignin
LN-PCL: lignin-grafted-polycaprolactone
MRDT: maximum rate of degradation temperature
MTZ: metribuzin
NMR: nuclear magnetic resonance
OH: hydroxyl group
PCL: poly­(ε-caprolactone)
ROP: ring-opening polymerization
Sn­(Oct)_2_: stannous 2-ethyl hexanoate
*T*_c_: crystallization temperature
TEM: transmission electron microscopy
TGA: thermogravimetric analyses
*T*_m_: melting temperature
*X*_c_ (%): crystallinity.
