# Supplementary material for: Multifunctional PCL/Lignin-PCL Composite Films for Delivery of Atrazine and Metribuzin for Sustainable Agriculture Applications
Source: ACS Agric Sci Technol. 2025 Jun 18;5(7):1351–61. doi: 10.1021/acsagscitech.5c00081 (PMC12284856; doi:10.1021/acsagscitech.5c00081)
Supplement: Supplementary file 1 [file as5c00081_si_001.pdf]

Supporting Information

Multifunctional PCL/lignin-PCL composite films for delivery of atrazine and metribuzin for sustainable agriculture applications

*Alvaro G. Garcia[1], Omar E. Mendez[1], Fannyuy V. Kewir[1], Gabriel D. Patterson[2], Artur Klamczynksi[2], Onu Onu Olughu[2], Carlos E. Astete[1], James D. McManus[2], Cristina M. Sabliov[1] **

[1]Biological & Agricultural Engineering, Louisiana State University and LSU Ag Center, Baton Rouge, Louisiana 70803, United States

[2] Bioproducts Research Unit, WRRC, ARS-USDA, Albany, California 94710, United States

*Corresponding author, csabliov@lsu.edu

Table of Contents

Figure S1. FT-IR spectra of 3100-3800 cm^{-1} region for PCL films that contain four different types of filler: LN and LN-PCL with three different DPs (26, 57, and 101). 3

Figure S2. FT-IR spectra of 1000-1400 cm^{-1} region for PCL films that contain four different types of filler: LN and LN-PCL with three different DPs (26, 57, and 101). 3

Figure S3. FT-IR spectra of 3150-3800 cm^{-1} region for MTZ-loaded PCL films that contain four different types of filler: LN and LN-PCL with three different DPs (26, 57, and 101). 4

Figure S4. FT-IR spectra of 1550-1750 cm^{-1} region for MTZ-loaded PCL films that contain four different types of filler: LN and LN-PCL with three different DPs (26, 57, and 101). 4

Figure S5. FT-IR spectra of 2800-3400 cm^{-1} region for ATZ-loaded PCL films that contain four different types of filler: LN and LN-PCL with three different DPs (26, 57, and 101). 5

Figure S6. FT-IR spectra of 1450-1900 cm^{-1} region for ATZ-loaded PCL films that contain four different types of filler: LN and LN-PCL with three different DPs (26, 57, and 101). 5

Figure S7. FT-IR spectra of 1050-1300 cm^{-1} region for ATZ-loaded PCL films that contain four different types of filler: LN and LN-PCL with three different DPs (26, 57, and 101). 6

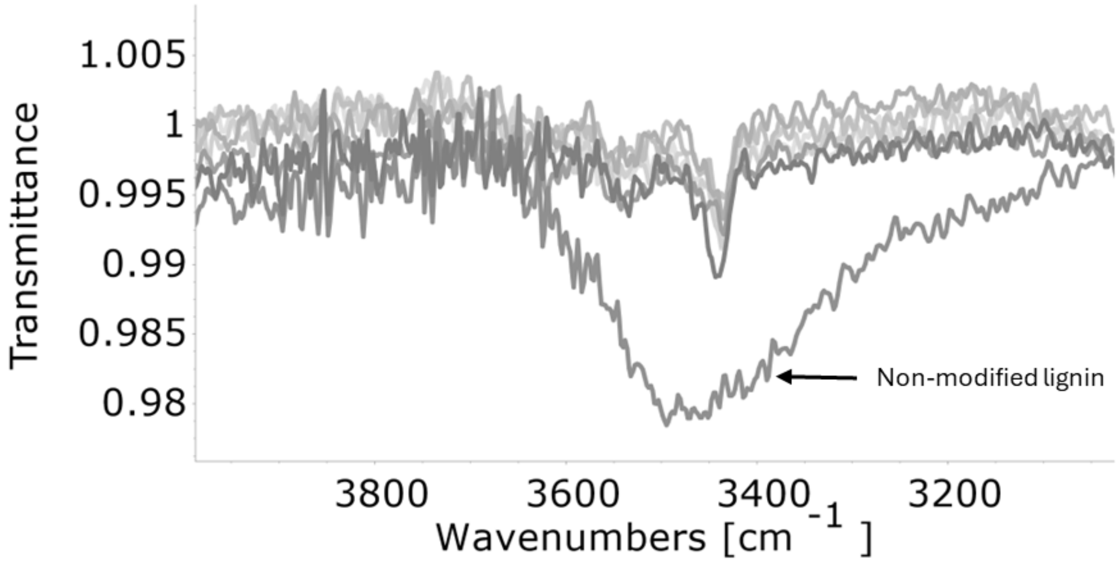

Figure S1. FT-IR spectra of 3100-3800 cm^{-1} region for PCL films that contain four different types of filler: LN and LN-PCL with three different DPs (26, 57, and 101). The spectrum of neat lignin and PCL_{103} is shown for comparison.

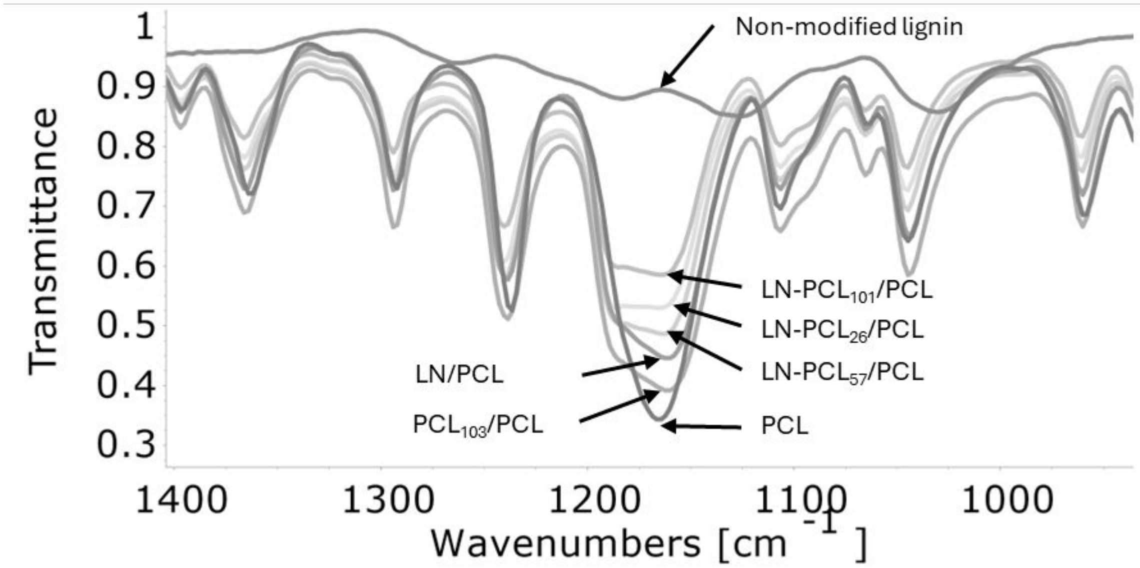

Figure S2. FT-IR spectra of 1000-1400 cm^{-1} region for PCL films that contain four different types of filler: LN and LN-PCL with three different DPs (26, 57, and 101). The spectrum of neat lignin and PCL_{103} is shown for comparison.

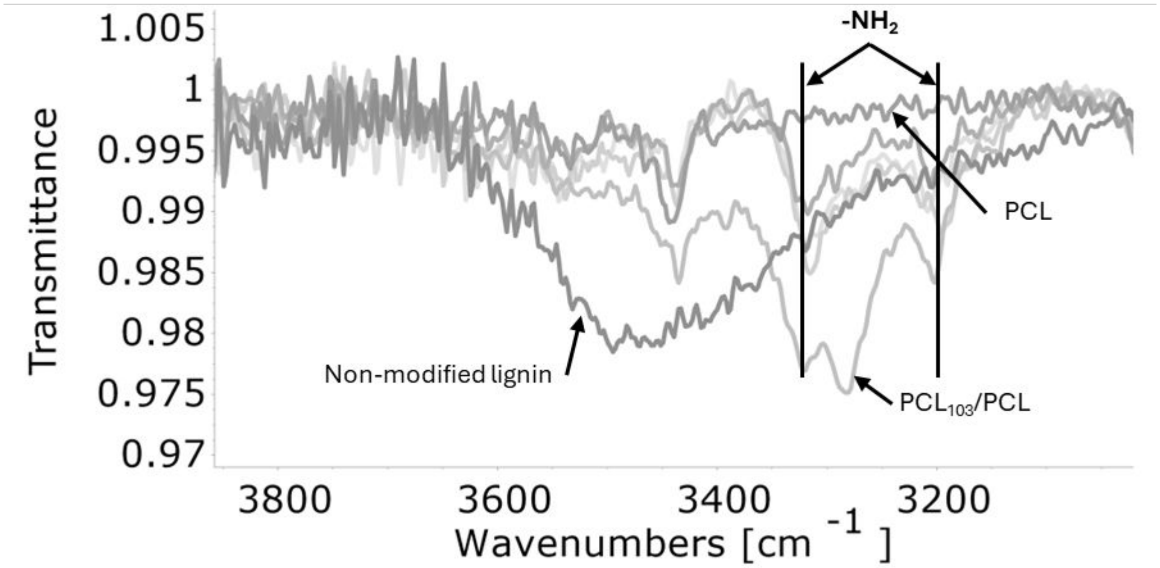

Figure S3. FT-IR spectra of 3150-3800 cm^{-1} region for MTZ-loaded PCL films that contain four different types of filler: LN and LN-PCL with three different DPs (26, 57, and 101). The spectrum of neat lignin and PCL_{103} is shown for comparison.

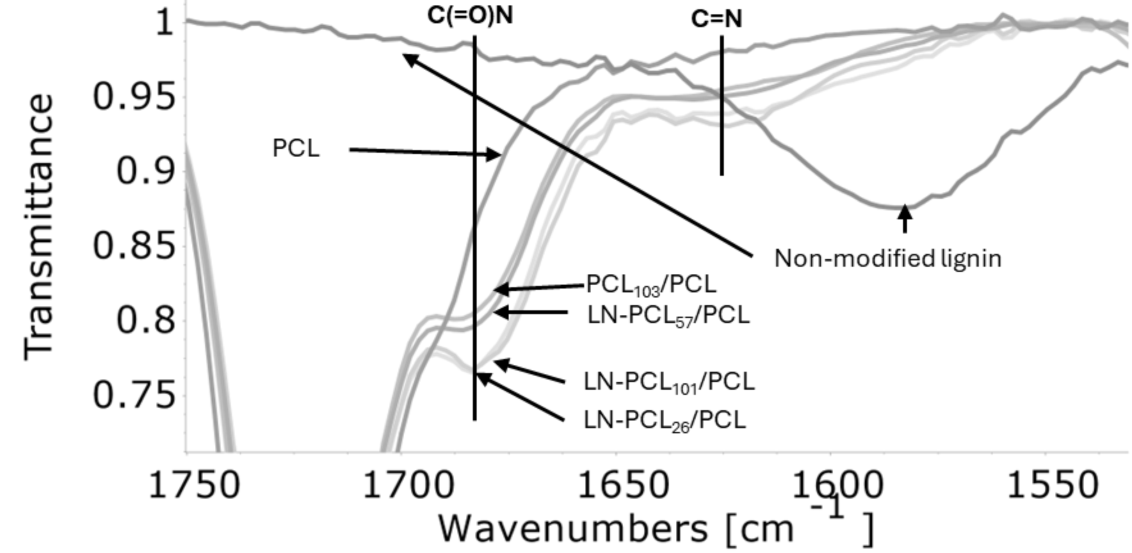

Figure S4. FT-IR spectra of 1550-1750 cm^{-1} region for MTZ-loaded PCL films that contain four different types of filler: LN and LN-PCL with three different DPs (26, 57, and 101). The spectrum of neat lignin and PCL_{103} is shown for comparison.

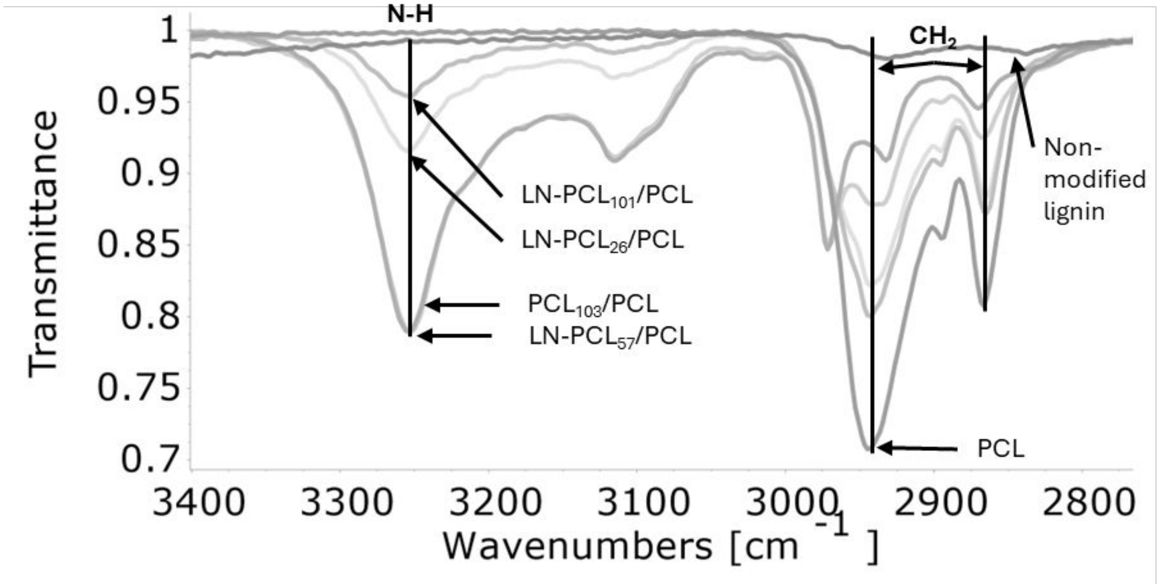

Figure S5. FT-IR spectra of 2800-3400 cm^{-1} region for ATZ-loaded PCL films that contain four different types of filler: LN and LN-PCL with three different DPs (26, 57, and 101). The spectrum of neat lignin and PCL_{103} is shown for comparison.

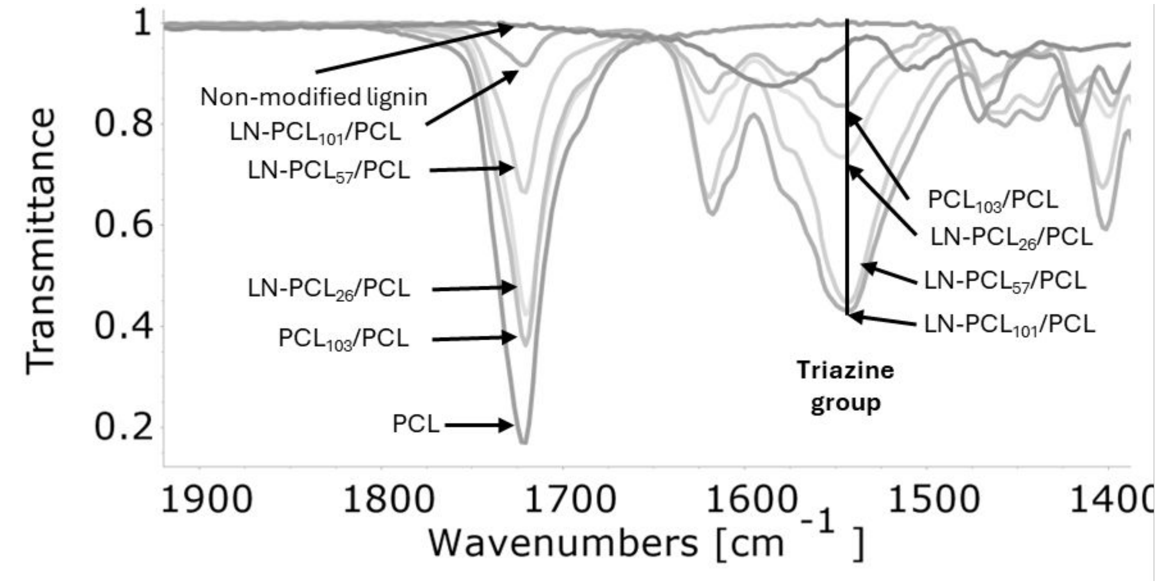

Figure S6. FT-IR spectra of 1450-1900 cm^{-1} region for ATZ-loaded PCL films that contain four different types of filler: LN and LN-PCL with three different DPs (26, 57, and 101). The spectrum of neat lignin and PCL_{103} is shown for comparison.

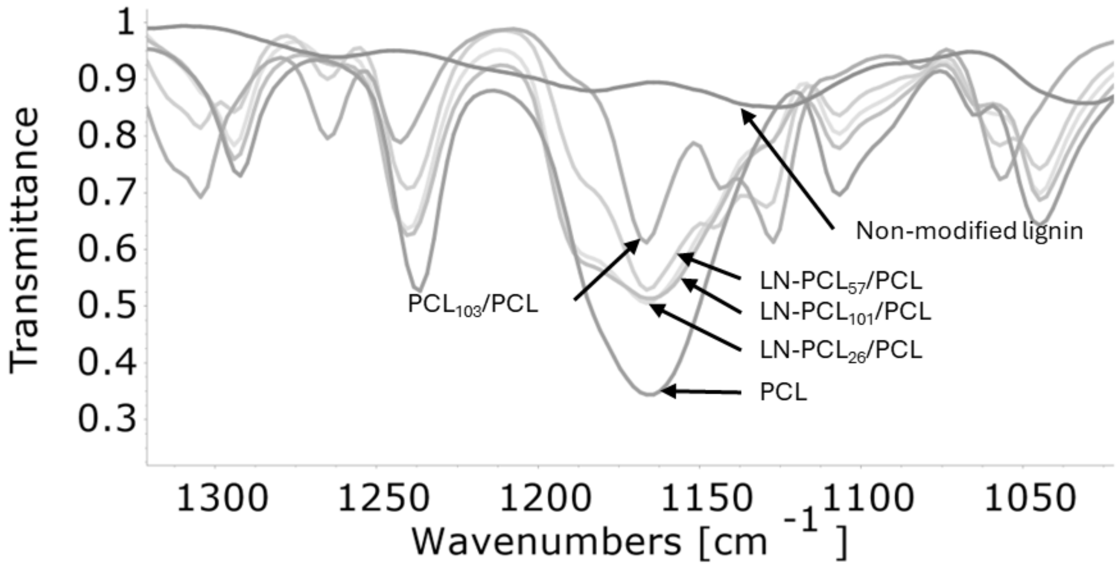

Figure S7. FT-IR spectra of 1050-1300 cm^{-1} region for ATZ-loaded PCL films that contain four different types of filler: LN and LN-PCL with three different DPs (26, 57, and 101). The spectrum of neat lignin and PCL_{103} is shown for comparison.
